# Outcomes of Middle Cardiac Vein Arterialization via Internal Mammary/Thoracic Artery Anastomosis

**DOI:** 10.1371/journal.pone.0080963

**Published:** 2013-11-20

**Authors:** Yang Yu, Hai-tao Li, Ming-xin Gao, Fan Zhang, Cheng-xiong Gu

**Affiliations:** Department of Cardiac Surgery, Beijing An Zhen Hospital, Capital Medical University, Beijing, China; Northwestern University, United States of America

## Abstract

**Objective:**

Cardiac vein arterialization is seldom applied for treating right coronary artery disease. This study aimed to improve outcomes of cardiac vein arterialization in a porcine model using intramammary artery anastomosis.

**Methods:**

A chronic, stenotic coronary artery model was established in 12 of 14 Chinese experimental miniature pigs of either sex, which were randomly divided into equal control (n = 6) and experimental (n = 6) groups. In experimental animals, blood flow was reconstructed in the right coronary artery using intramammary artery. Arterialization involved dissection of right internal mammary artery from bifurcation to apex of thorax followed by end-to-side anastomosis of internal mammary artery and middle cardiac vein plus posterior descending branch of right coronary artery. Intraoperative heart rate was maintained at 110 beats/min. Graft flow assessment and echocardiography were performed when blood pressure and heart rate normalized.

**Results:**

The experimental group had significantly higher mean endocardial and epicardial blood flow postoperatively than control group (mean endocardial blood flow: 0.37 vs. 0.14 ml/(g*min), p<0.001; mean epicardial blood flow: 0.29 vs. 0.22, p = 0.014). Transmural blood flow was also higher in experimental group than in control group (0.33 vs. 0.19, p<0.001); ejection fraction increased from 0.46% at baseline to 0.51% (p = 0.0038) at 6 hours postoperatively, and mean blood flow of internal mammary artery was 44.50, perfusion index 0.73 at postoperative 6 months, 43.33 and 0.80 at 3 months.

**Conclusion:**

Successful cardiac vein arterialization via intramammary artery in a porcine model suggests that this may be a viable method for reconstructing blood flow in chronic, severe coronary artery disease.

## Introduction

Currently, most patients with chronic or acute (post-myocardial infarct) coronary artery disease (CAD) are treated with coronary artery bypass grafting (CABG) or percutaneous coronary intervention (PCI). Long-term outcomes of these procedures are generally favorable. The advent of antiplatelet therapy and drug-eluting stents have increased the success rates of PCI[1]. In a recent study, six-year follow-up of PCI with drug-eluting stent implantation for left main coronary artery stenosis revealed acceptable rates of cardiac death, myocardial infarction and stent thrombosis[2]. However, patients with refractory angina, including severe diffuse coronary artery stenosis, small caliber coronary artery, and a history of repeated surgeries, cannot undergo CABG or PTCA, and these patients account for 12% to 15% of patients requiring myocardial revascularization therapy[3]. Surgeons and scholars have devised other techniques, including transmyocardial laser revascularization (TMLR)[4], pro-angiogenic gene therapy[5], cardiac denervation surgery, and endarterectomy combined with CABG[6]. Specific limitations preclude wide acceptance of these methods. TMLR is associated with high early mortality (6.8% 30-day mortality rate)[7]. Molecular therapies via the coronary artery and cardiac surgery have both failed to support revascularization of the myocardium[8]. Cardiac denervation surgery only alleviates the degree of angina short-term[9]. Endarterectomy is not suitable for thin blood vessels with lesions, blood vessels with severe distal lesions, or blood vessels with immature plaques[10.] Another method is percutaneous in situ coronary venous arterialization (PICVA), which is difficult to implement when the coronary artery has severe diffuse lesions[11]. Ventricle-to-coronary vein bypass (VPASS) may also be applied, however, this procedure decreases pressure of the coronary venous system to such an extent that the blood flow of retrograde perfusion is reduced, which may easily progress to inadequate perfusion[12].

In the 1970s, based on the idea that atherosclerosis does not affect the venous system, the concept of selective coronary vein arterialization was proposed for patients not able to receive CABG or PCI[13], [14]. Revascularization of the myocardium by coronary venous bypass grafting (CVBG) was performed in animal models using healthy veins to achieve retrograde flow into the capillary vascular bed[14], [15]. Although surgeons reported that 93% of patients receiving planned CVBG survived, and 22 of 25 evaluated patients had symptomatic improvement[16], the procedure is still not often performed. Møller et al. (2007) re-explored CVBG in an animal model using less invasive (off-pump) methods for revascularization and modern equipment for monitoring[17]. Results indicated that CVBG was an effective way to revascularize the myocardium after acute myocardial events when CABG and PCI were not appropriate. Good clinical results were also reported for a challenging case in which a patient with unstable angina and an aberrant left coronary system received myocardial revascularization by means of venous grafting[18].

Currently, however, cardiac vein arterialization is not a major treatment for CAD because procedural aspects, graft materials and outcomes are still being questioned. For example, common use of the great saphenous vein as the bypass graft is complicated by the inability of the coronary vein to tolerate high arterial pressure for a long period of time, which may result in hemorrhage, edema, endometrial hyperplasia or even occlusion of the coronary venous system[19]. However, the internal mammary artery, which functions as a muscular tube, is able to respond to changes in blood flow, and pressure in the lumen of the internal mammary artery is low, which helps to buffer the pressure in the coronary venous system. Therefore, we hypothesized that by replacing the saphenous vein as the usual choice for arterialization with the internal mammary artery as the graft material, we could successfully reconstruct blood flow in severe, diffuse coronary artery disease, possibly improving response to blood flow changes and reducing pressure in the coronary venous system after bypass grafting, thereby achieving successful outcomes. The purpose of this study was to reduce potential complications of cardiac vein arterialization in a porcine model of chronic, stenotic CAD using intramammary artery anastomosis. Applying the internal mammary artery as the graft material may be especially suitable for the specific patient population with chronic CAD, providing compensation of the collateral circulation with a synergistic effect on retrograde flow from the arterialized vein.

## Materials and Methods

### Animal model construction

All animal procedures were approved by the Ethics Committee of Beijing An Zhen Hospital, Capital Medical University, and the protocols followed the ethical guidelines of this committee. All animals were allowed free access to water and diet.

Experimental animals: Chinese experimental miniature pigs were purchased from Beijing Agriculture University (n = 14) and were of either sex with an average body weight of 40±5 kg. They were fed a high cholesterol diet for 4 weeks (main ingredients were: 10.0% egg yolk powder, 6.0% cholesterol, 8.0% lard, 8.0% peanut oil, and 1.2% salt) and then underwent endarterectomy of the right coronary artery. Aspirin 325 mg was administered one day before surgery and the animals were fasted 8 hours before surgery. Anesthesia involved intramuscular injection of diazepam (1.5 mg/kg) and ketamine (3.5 mg/kg) 15 min before surgery. Endotracheal intubation was then performed and the ventilator (Vela, Viasys Healthcare, San Diego, USA) was set at 15–20 times/min to assist breathing as close as possible to normal physiological status. Intraoperative anesthesia was maintained with diazepam (0.5 mg/kg), and continuous ECG monitoring was carried out. During intervention, fentanyl (0.75 mg/kg/45 min, IV) was used as an analgesic and 1.5 mg/kg atracurium besylate intravenous bolus was administered to induce muscular relaxation. Marginal ear vein was punctured to establish intravenous access. Right femoral artery puncture was performed, and the femoral artery sheath was rinsed with heparin and inserted via guide wire (St. Jude Medical, St. Paul, MN, USA). Heparin 1000 U/kg was injected, and heparin 2000 U was injected intravenously once every hour. Coronary angiography was performed and 6F JR catheter (Cordis Corporation, Holland) was sent to the middle section of the ascending aorta along the arterial sheath under digital subtraction angiography (DSA). The catheter was pushed slowly to the opening of the right coronary artery and 76% diatrizoate was injected. A 6F JR catheter was used for right coronary angiography. Balloon (Medtronic, Minneapolis, MN, USA) expansion and stretching were done first to send the 7F JR guiding catheter to the opening of the right coronary artery through the femoral artery sheath, and then the guide wire was sent to the distal segment of the right coronary artery through the guiding catheter. The 2.5 mm or 3.0 mm balloon catheter (selected according to right coronary artery diameter) was inserted along the guidewire into the distal, middle-distal and middle segments of the right coronary artery in succession to perform balloon expansion. The ratio of the balloon diameter to the coronary artery diameter was 1.3∶1, and the balloon inflation pressure was 4–6 atm. In order to cause mechanical exfoliation of the arterial intima, each segment of the right coronary artery was expanded by the balloon and stretched 3 times, for 30 seconds each time. The interval was 60 seconds. The contrast agent was aspirated, creating negative pressure in the balloon before it was withdrawn. The pigs were then raised in individual cages and continued to receive the high-cholesterol diet. Intramuscular injection of 4 million U penicillin was administered for 3 days to prevent infection. After 8 weeks, coronary angiography was performed via the femoral artery on the opposite side (left) to observe the coronary artery stenosis. Animals with severe diffuse right coronary artery stenosis (degree of stenosis ≥90%; stenosis length >1 cm) were suitable for undergoing subsequent experiments.

Of 14 animals, severe diffuse right coronary artery stenosis was successfully modeled in 12 animals. They were randomly and equally divided into an experimental group that would receive cardiac vein arterialization and a control group that would receive exploratory thoracotomy only. Postoperative evaluation was conducted in both groups and results were compared.

### Cardiac vein arterialization

**Figure 1:**
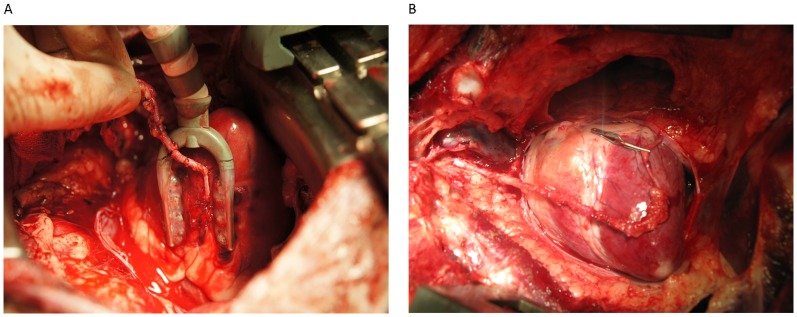
Dissection of the right internal mammary artery and end-to-side anastomosis of the internal mammary arter and middle cardiac vein. (A)The right internal mammary artery was dissected completely. (B) The proximal end of the middle cardiac vein was ligated. End-to-side anastomosis of the internal mammary artery and middle cardiac vein was performed.

After anesthesia and median sternotomy and thoracotomy, the right internal mammary artery was dissected for later use. In order to ensure sufficient length, the right internal mammary artery was dissected from the bifurcation to the apex of the thorax (Figure 1A). The proximal end of the middle cardiac vein was ligated using silk thread. The heart rate was maintained to prevent blood from returning to the coronary sinus via the normal path. An 8-0 prolene suture was used for end-to-side anastomosis (Figure 1B) of the internal mammary artery and the middle cardiac vein accompanied by the posterior descending branch of the right coronary artery. Preoperative echocardiography was performed, and intraoperative continuous ECG monitoring (Vela, Viasys Healthcare, San Diego, USA) was adopted so that the intraoperative heart rate was maintained at about 110 beats/min. After surgery, the anastomotic stoma was examined to ensure that no bleeding was present. Graft flow assessment and echocardiography were performed when blood pressure and heart rate were normal.

### Graft flow assessment and echocardiography

Graft flow assessment was performed using the Medistim Veri-Q Flow Meter System with QuickFit TTFM probe (Medistim,Oslo, Norway). Echocardiography (HP SONOS 5500) was used to calculate the pre-operative and postoperative ejection fraction (EF), E/A ratio (E: peak early diastolic transmitral flow velocity; A: atrial peak transmitral flow velocity). The ultrasound section was a parasternal left ventricular long axis view.

### Determination of myocardial blood flow

Non-radioactive colored microsphere method (Triton Technology, San Clemente, CA, USA) was used to detect the blood flow of various myocardial layers, including subendocardial, subepicardial, and transmural blood flow. The animals received injections of 2 ml yellow non-radioactive colored microspheres (diameter 15±1 µm, each milliliter contains 300,000 microspheres) through the aortic root and the internal mammary artery graft, respectively. At the same time, a fixed speed pump was used to draw the reference blood samples via the common carotid artery catheter at a rate of 7.64 mL/min for a total of two minutes. The animals were sacrificed with an intravenous overdose infusion of potassium chloride solution and 4 g samples of the right ventricular myocardium were collected from the two groups. Samples were divided into equal-sized endocardial and epicardial myocardial tissue blocks and sent for pathologic examination. Each tissue block weighed about 2 g. The microsphere dye extraction reagents (Triton Technology) were used to extract the dye. A spectrophotometer was used, and a filter with a 10 nm bandwidth and center absorption wavelength of 450 nm was selected to measure the optical density of the dye extract of yellow microspheres. The unit myocardial blood flow (MBF) was calculated. The MBF ml/(g.min)  =  (ODm×Qr)/(ODr×Wm), of which MBF is the unit myocardial blood flow, Qr is the drawing velocity of the reference blood samples, and ODr and ODM are the optical density of the reference blood samples and the microsphere dye extract of the myocardial tissue, respectively. Wm is the weight of the myocardial tissue block.

### Statistical analysis

Data are presented as mean with standard deviation (mean±SD). Comparisons between control and SCVBG groups were performed by the independent two samples t-test. Comparisons for the changes of the three time points from baseline to post-surgery within groups were performed by repeated-measures analysis of variance, with Bonferroni post-hoc tests. Statistical hypothesis tests were two-sided. A p-value of <0.05 was considered as statistically significant. Statistical analyses were performed using SPSS15.0 (SPSS Inc, Chicago, IL, USA).

## Results

**Figure 2:**
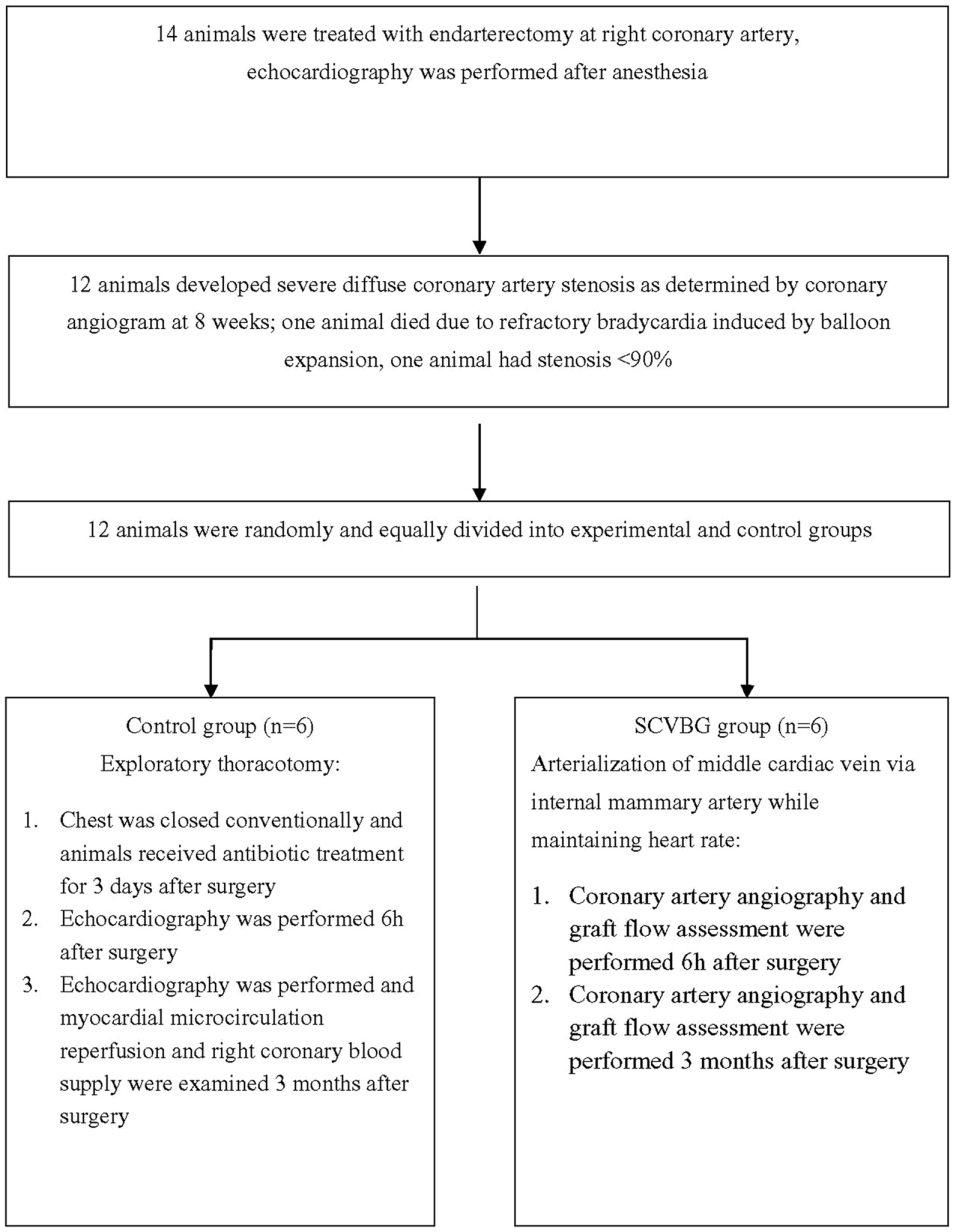
Animal model construction.

A porcine model of severe diffuse coronary artery stenosis was successfully established in 12 of 14 animals. One animal died due to refractory bradycardia induced by balloon expansion and one animal failed due to a stenosis rate of <90%. The 12 animals were equally and randomly divided into control and selective coronary vein bypass graft (SCVBG) groups. Control group animals received exploratory thoracotomy and experimental animals received arterialization of the middle cardiac vein via the internal mammary artery while maintaining heart rate. (Figure 2)

At baseline, animals' weights and diffuse coronary artery stenosis rates were comparable in control and SCVBG groups. After surgery, significantly higher mean blood flow was observed in the endocardium and epicardium of the SCVBG group compared to that in the control group (mean endocardial blood flow: 0.37 vs. 0.14 ml/(g*min), p<0.001; mean epicardial blood flow: 0.29 vs. 0.22, p = 0.014). The transmural blood flow was also higher in the SCVBG group than in the control group (0.33 vs. 0.19, p<0.001). In addition, the SCVBG animals had mean internal mammary artery blood flow of 44.50 and 43.33 and PI of 0.73 and 0.80 at 6 hours and at 3 months after surgery. No significant change was observed in mean blood flow of internal mammary artery and PI between 6 hours and 3 months after surgery. No significant differences were found in baseline EF and E/A between the two groups. (Table 1)

**Table 1:** Pre- and post-operative comparisons between experimental and control groups.

		Control group (n = 6)	SCVBG group (n = 6)	P-value
**Baseline**				
Weight (kg)		43.33±2.34	43.17±2.93	0.915
Diffuse coronary artery stenosis rate (%)		96.17±4.79	94.83±5.31	0.658
**Postoperative ^a^**				
Mean endocardial blood flow (ml/(g*min))		0.14±0.04	0.37±0.06	<0.001*
Mean epicardial blood flow (ml/(g*min))		0.22±0.03	0.29±0.05	0.014*
Transmural blood flow ml/(g*min)		0.19±0.03	0.33±0.05	<0.001*
Mean blood flow of internal mammary artery	in 6 hours	-	44.50±5.86	
	in 3 months	-	43.33±5.01	
PI	in 6 hours	-	0.73±0.14	
	in 3 months	-	0.80±0.14	

PI, percutaneous intervention; SCVBG, selective coronary vein bypass graft.*indicates significant difference between groups. ^a^Control group: exploratory thoracotomy; SCVBG group: Arterialization of middle cardiac vein was implemented via the internal mammary artery while maintaining heart rate.

**Figure 3:**
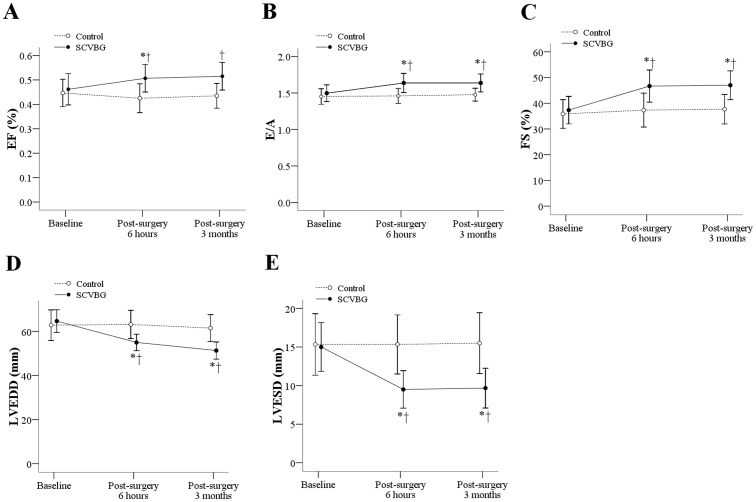
Changes in echocardiographic parameters from baseline to postoperative 6 months. (A) EF, (B) E/A ratio, (C) LVEDD, (D) LVESD, (E) FS. Data are presented as mean and standard deviation. In SCVBG group, EF was significantly higher than at baseline (p = 0.0038) than at postoperative 6 hours; E/A ratios were significantly higher than baseline (p = 0.0067 and 0.009) at postoperative 6 hours and 3 months. EF, E/A, and FS in SCVBG group were significantly higher than in control group at postoperative 6 hours and 3 months (EF: p = 0.0341 and p = 0.0271; E/A ratio: p = 0.0269 and p = 0.0268; FS: p = 0.030 and p = 0.017). LVEDD and LVESD in SCVBG group were significantly lower than in control group at postoperative 6 hours and 3 months (LVEDD: p = 0.027 and 0.006; LVESD: p = 0.016 and p = 0.019). EF, ejection fraction; E/A, E/A ratio, E: the peak early diastolic transmitral flow velocity; A: atrial peak transmitral flow velocity; SCVBG, selective coronary vein bypass graft. *indicates a significant difference compared to baseline within group. †indicates a significant difference between control and SCVBG groups at the specific time point(s).

At 6 hours and at 3 months postoperatively, significantly higher EF, E/A ratio, and FS were observed in the experimental group than in the control group (E/F: 6 hours: 0.51 vs. 0.43, p = 0.0341; 3 months: 0.52 vs. 0.44, p = 0.0271; E/A ratio: 6 hours: 1.64 vs. 1.46, p = 0.0269; 3 months: 1.64 vs. 1.48, p = 0.0268; FS: 6 hours: 46.67 vs. 37.33, p = 0.03; 3 months: 47.0 vs. 37.67, p = 0.017). Also, significantly lower postoperative LVEDD and LVESD were observed in the experimental group than in the control group (LVEDD: 6 hours: 55.0 vs. 63.17, p = 0.027, 3 months: 51.33 vs. 61.5, p = 0.006; LVESD: 6 hours: 9.5 vs. 15.33, p = 0.016; 3 months: 9.67 vs. 15.5, p = 0.019). In addition, a significant increase of EF from 0.46% at baseline to 0.51% at post-operative 6 hours (p = 0.0038), significant increases of E/A ratio from 1.5 at baseline to 1.64 at post-surgery 6 hours and 3 months (p = 0.0067 and p = 0.009), and significant increases of FS from 37.33 at baseline to 46.67 and 47.0 at post-surgery 6 hours and 3 months were observed in the experimental group (p = 0.013 and p = 0.011). Significant decreases of LVEDD from 64.67 mm at baseline to 55.0 and 51.33 mm at post-operative 6 hours and 3 months were observed (p<0.001 and p = 0.001). Significant decreases of LVESD from 15.0 mm at baseline to 9.5 and 9.67 mm at post-operative 6 hours and 3 months were observed (p = 0.03 and p = 0.04). (Figure 3)

**Figure 4:**
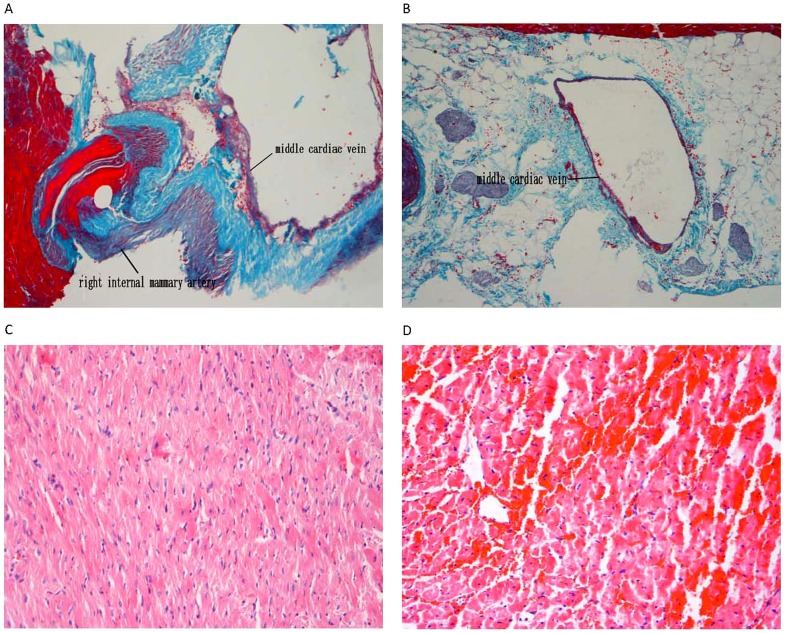
Histological changes in 3-month follow-up of experimental and control groups. (A) Experimental group—three months after ligation, no thickening or fibrosis was found near the ligation site. (B) Control group, middle cardiac vein. (C) Experimental group, region of ischemia. (D) Control group, region of ischemia, showing disordered arrangement of myocytes with cell swelling and karyorrhexis.

Additional images are provided showing 3-month follow-up of experimental and control groups, including the ligation site fibrosis status, cardiac wall and myocyte arrangement. (Figures 4 A, B, C, D)

## Discussion

The author successfully established a chronic, stenotic coronary artery porcine model focused on the right coronary artery, and blood flow was reconstructed using the intramammary artery for arterialization. Evaluation of this modified SCVBG procedure revealed that the SCVBG group had significantly higher mean blood flow in the endocardium and epicardium compared to controls and the transmural blood flow was also higher. At two time points, 6 hours and 3 months postoperatively, the mean blood flow of the internal mammary artery was 44.50 and 43.33, respectively, and PI was 0.73 and 0.80, respectively. Significantly higher EF and E/A ratios were also observed in the experimental group at these time points. Our satisfactory results in a porcine model show that revascularization is not only possible but that, as we had hypothesized, replacing the saphenous vein with the internal mammary artery as the graft material allows effective reconstruction of blood flow in severe, diffuse coronary artery disease, avoiding potential complications commonly associated with bypass grafting.

We are not the only investigators to construct an animal model of CAD and to substitute the mammary artery for SCVBG. Møller et al. constructed a porcine model of CAD, focusing on the left coronary vein, and conducted selective retrograde coronary vein bypass grafting using an off-pump technique, applying various modern methods of monitoring the physiological effects of this procedure[17]. In their procedure, the left internal mammary artery was anastomosed to the left anterior descending coronary vein. Results showed that the myocardium could be nourished to a certain extent with this procedure, preventing death even though varying amounts of ischemia remained. The authors suggested that SCVBG was a viable way to revascularize the myocardium after acute ischemic events when CABG and PCI were not appropriate treatments[17].

Given the encouraging results from our study and that of Møller et al., we must look at the clinical importance of a chronic CAD model, and the possible reasons for unsatisfactory results of previous studies of cardiac vein arterialization. In previous studies of selective retrograde coronary venous perfusion, animal models of acute myocardial infarction were established by ligating the initial segment of the anterior descending branch[20], [21]. Animals receiving selective retroperfusion with proximal ligation of the vena cordis magna had significantly better hemodynamic performance and long-term survival, suggesting that venous retroperfusion can effectively achieve long-term survival after acute occlusion of the left anterior descending artery in a porcine model[21]. However, the presence of a large area of acute myocardial infarction may easily lead to intractable ventricular fibrillation, resulting in a low survival rate of the animals. In order to improve the success rate of myocardial infarction model construction, the distal segment of the first diagonal branch can be ligated to reduce the range of myocardial ischemia. This may reduce the success rate of coronary venous arterialization due to the presence of competition from the antegrade blood flow[20]. In addition, the acute myocardial infarction model cannot simulate the pathophysiological state of ischemic myocardium in clinical practice[21]. A chronic myocardial ischemia model reduces the occurrence of fatal ventricular fibrillation, and, more importantly, is more in line with the pathophysiological process of coronary stenosis. The volume of the antegrade blood flow in the coronary artery with diffuse stenosis is small, resulting in less impact on retrograde perfusion.

Models of chronic myocardial ischemia are mainly established through chronic mechanical compression of the coronary artery, which requires complex open chest surgery and causes great trauma to the animals, who cannot be raised after surgery. This study focused on the right coronary artery for these reasons. In clinical practice, sequential coronary artery bypass grafting is usually adopted, but since the right coronary artery is the terminal branch of the sequential graft, the patency of the right coronary artery bypass graft will directly affect the patency of the entire sequential graft. We applied balloon expansion and stretching to establish a chronic diffuse right coronary artery stenosis model in pigs. A great number of choices are available for experimental animals for model building. Rabbits fed with a high cholesterol diet can develop atherosclerosis, and balloon expansion can cause restenosis lesions, although rabbits are small for difficult heart surgeries. A dog model usually has poor stability because collateral circulation forms too easily in dogs after myocardial ischemia. Nevertheless, Ido et al. (2001) showed that coronary sinus occlusion enhanced coronary collateral flow and reduced subendocardial ischemia in a dog model[22]. Resetar et al. performed selective arterialization in a sheep model under circumstances of significant artherosclerosis not amenable to revascularization of the coronary artery due to combined cardiac micro- and macroangiopathy[23]. Myocardial ischemia was confirmed by a significant decrease in cardiac output, stroke volume and mean arterial blood pressure, indicating successful retrograde arterialization in this model. Pigs, too, have many advantages in constructing an animal model of chronic myocardial ischemia. Their cardiovascular morphology, physiology, and atherosclerosis susceptibility are closest to that of humans. The angiogenesis capacity of pigs is exceptionally limited after myocardial ischemia, creating good model stability. Additionally, balloon expansion and stretching performed in the porcine coronary artery can result in postoperative traumatic stenosis, which is very similar to the pathological process in humans. Harig et al. investigated arterialization of cardiac veins in an experimental long-term porcine model, finding that venous retroperfusion was effective in achieving long-term survival after acute occlusion of the left anterior descending artery in the pig model[21].

A recent report by Yu et al. (2011) described performance of off-pump coronary artery bypass (OPCAB) surgery in 17 patients as a less invasive way to achieve myocardial revascularization. The procedure allows performance of sequential bilateral mammary artery grafting combined with selective arterialization of the coronary venous system; ischemia was reduced in all patients and substantial improvement was seen in cardiac function and quality of life, suggesting a better long-term prognosis and the likelihood of extensive application of the technique[24]. In contrast, earlier human studies suggested a lack of durability of arterialization of coronary vein and expressed doubts that the technique was useful for revascularization of the ischemic myocardium[25].

The limitations of coronary angiography must be considered when evaluating results of the present study. In depth studies on the hemodynamics and pathophysiology of the coronary artery have shown that coronary angiography as a single modality is not a unique standard able to guide the performance of vascular reconstructive surgery. Coronary angiography fails to completely evaluate the physiological functions of the stenotic coronary artery and therefore we cannot determine whether the stenotic coronary artery may cause evident myocardial ischemia by applying coronary angiography alone. Fractional flow reserve (FFR), however, may provide additional evidence on functional changes in the stenotic coronary artery based on evaluation by coronary angiography and also provide evidence for therapeutic strategies [26]. An FFR of ≤0.8 suggests the association between coronary stenosis and myocardial ischemia, and vascular reconstructive surgery is required in this circumstance. On the other hand, an FFR of >0.8 suggests that the lesioned coronary artery may not cause evident myocardial ischemia and special treatment is not required [27]. However, FFR is mainly used in the evaluation of threshold lesions of the coronary artery. In the present study, the coronary stenosis in the CAD model was severe and FFR detection was not needed. However, performance of myocardial perfusion SPECT imaging may have been ideal to determine the site of myocardial ischemia, quantify the myocardial infarction and identify the survived myocardium, as previously demonstrated [28]. After establishing the presence of severe coronary stenosis, it is necessary to evaluate whether vascular reconstructive surgery is needed. When either stunned ischemic myocardium or hibernating myocardium are absent, vascular reconstructive surgery is not needed, and patients with such coronary lesions should be excluded from study to avoid biased results. While performance of SPECT imaging was indicated in the present study, myocardial perfusion SPECT imaging is not yet available in our center due to lack of the device. Instead, we used non-radioactive colored microspheres to detect the reperfusion of myocardial microcirculation. Results showed that the blood flow of the ischemic myocardium increased in the experimental group when compared to preoperative blood flow after arterialization of coronary veins, accompanied by improvement of post-operative cardiac function. These parameters indirectly indicate the presence of stunned ischemic myocardium or hibernating myocardium. Our future studies will involve detection of the physiological functions of the coronary artery based on the points discussed above.

The present study has certain other limitations. First, the sample was small with only a limited number of animals in each group, which may affect analysis of results. Further, because controls were not subject to revascularization, results cannot be compared to other forms of arterialization in the myocardial venous system. We also did not have data to indicate whether angiogenesis was improved in the ischemic myocardium. However, our results indicate the potential benefit of direct revascularization and suggest that further study is needed to confirm this benefit in clinical practice.

## Conclusions

In conclusion, successful cardiac vein arterialization via intramammary artery anastomosis in a porcine model suggests that this may be a viable method for reconstructing blood flow in chronic, severe coronary artery disease. The methods we have employed were shown to be effective and to avoid complications associated with bypass grafting in CAD; they may be suitable for chronic CAD patients who require compensation of collateral circulation, which may have a synergistic effect on retrograde flow from the arterialized coronary vein. This alternate technique could be especially relevant for patients with severe, diffuse coronary artery disease whose arteries are inaccessible for CABG and PCI or who have had previous failed surgeries or restenosis.
